# Cytochrome P450 single nucleotide polymorphisms in an indigenous Tanzanian population: a concern about the metabolism of artemisinin-based combinations

**DOI:** 10.1186/1475-2875-13-420

**Published:** 2014-11-03

**Authors:** Karol J Marwa, Theresa Schmidt, Maria Sjögren, Omary MS Minzi, Erasmus Kamugisha, Göte Swedberg

**Affiliations:** Department of Pharmacology, Catholic University of Health and Allied Sciences, Mwanza, Tanzania; Institute of Medical Biochemistry and Microbiology, Uppsala University, Uppsala, Sweden; Unit of Pharmacology and Therapeutics, Muhimbili University of Health and Allied Health Sciences, Dar Es Salaam, Tanzania; Department of Biochemistry, Catholic University of Health and Allied Sciences, Mwanza, Tanzania

**Keywords:** Cytochrome P450, Artemisinin-based combination therapy, Poor metabolizers, Tanzania, *Plasmodium falciparum* malaria

## Abstract

**Background:**

Artemisinin-based combinations currently recommended for treatment of uncomplicated *Plasmodium falciparum* malaria in many countries of sub-Saharan Africa are substrates of CYP enzymes. The cytochrome enzyme system is responsible for metabolism of about 80-90% of clinically used drugs. It is, therefore, important to obtain the pharmacogenetics of the population in the region with respect to these combinations and thereby enable practitioners to predict treatment outcomes. The aim of this study was to detect and determine allelic frequencies of CYP2C8*2, CYP2C8*3, CYP3A4*1B, CYP3A5*3 and CYP2B6*6 variant alleles in a Tanzanian indigenous population.

**Methods:**

Genomic DNA extraction from blood obtained from 256 participants who escorted patients at Karume Health Centre in Mwanza Tanzania, was carried out using the Gene JET™ Genomic DNA purification kit (Thermo Scientific). Genotyping for the cytochrome P450 variant alleles was performed using predesigned primers. Amplification was done by PCR while differentiation between alleles was done by restriction fragment length polymorphism (PCR-RFLP) (for CYP2C8*2, CYP2C8*3) and sequencing (for CYP2B6*6, CYP3A5*3 and CYP3A4*1B).

**Results:**

CYP2C8*2, CYP2C8*3, CYP3A5*3, CYP3A4*1B and CYP2B6*6 variant allelic frequencies were found to be 19,10,16,78 and 36% respectively.

**Conclusion:**

Prevalence of CYP2C8*2, CYP3A5*3, CYP3A4*1B and CYP2B6*6 mutations in a Tanzanian population/subjects are common. The impact of these point mutations on the metabolism of anti-malarial drugs, particularly artemisinin-based combinations, and their potential drug-drug interactions (DDIs) needs to be further evaluated.

## Background

Prevention and treatment of malaria is mainly based on the use of drugs [1]. Treatment response in *Plasmodium falciparum* malaria depends on various factors such as parasite resistance, host natural immunity, drug quality and the pharmacokinetics of the administered drug [2]. Various studies have been carried out on molecular mechanisms for resistance in malaria chemotherapy [3]. However limited efforts have been made to determine genetic polymorphisms in cytochrome P450 enzymes, which may be associated with treatment failure (in extensive metabolizers) as a result of subtherapeutic drug concentrations. Cytochrome P450 (CYP) polymorphisms may also cause toxicity (in slow metabolizers) due to high drug concentrations and emergence or spread of drug resistance (in extensive metabolizers) as a result of subtherapeutic drug concentrations. Recent studies have also associated drug resistant selection with poor metabolizer (PM) phenotype.

The increasing use of artemisinin-based combinations as an effective treatment of resistant malaria demands determination of genetic polymorphisms in the metabolism of these drugs and its influence on pharmacokinetic profiles between individuals, adverse drug reactions and clinical outcome.

Polymorphisms in gene encoding for drug- metabolizing enzymes and transporters are associated with individual variation in drug response [4]. The frequency of variant alleles encoding for CYP families varies among populations according to race and ethnic background [5].

Cytochrome P450 metabolize about 80-90% of clinically used drugs [5–8]. About 40% of cytochrome P450 dependent-drug metabolism is due to polymorphic enzymes [9]. Polymorphisms occur in all members of the CYP2C subfamily, which are CYP2C8, CYP2C9, CYP2C18 and CYP2C19 [10, 11]. The genes for these subfamilies are located on chromosome 10q24 [12]. The role of CYP genes in metabolism varies significantly. For example, CYP2C has been shown to be responsible for metabolism of about 20% of clinically used drugs and endogenous substances [10].

CYP2C8 has been proven to make up 7% of the total CYP content of the liver [13] and plays a major role in the metabolism of many clinically available drugs [14–16] accounting for about 5% of the drugs used clinically [17]. The CYP2C8*2 allele has been reported to be common in Africans, but rare in Asians and Caucasians, whereas CYP2C8*3 has been reported to be common in Caucasians, but rare in Africans or Asians. The latter allele (CYP2C8*3) has been associated with a marked reduction in amodiaquine metabolism in Caucasian population [14].

CYP2B6 is located within the CYP2B gene cluster on chromosome 19 [18] and is one of the most polymorphic genes in humans [19]. This gene contributes 2-10% of the total CYP content [20]. CYP2B6*6 is more frequent in African descent than Caucasoids [21, 22] and is associated with raised plasma concentrations of antiretroviral (efavirenz, nevirapine) [19] and anti-malarial drugs (artemisinin and arteether) [1, 19].

Cytochrome P450 subfamily CYP3A is the most abundant CYP in the human liver and small intestine [23]. CYP3A4, which is part of CYP3A gene cluster, is involved in the metabolism of approximately 50% of clinically used drugs [5, 24].

CYP3A4 is widely considered to be the dominant CYP3A isoform, but this has been questioned lately when data have shown that the amount of CYP3A5 is much larger than previously assumed in individuals expressing this enzyme [25]. The most common single nucleotide polymorphism (SNP) within the CYP3A4 family is CYP3A4*1B [26], an A to G transition at 392 of the proximal promoter region of the gene. The CYP3A4*1B variant allele is shown to be associated with higher expression of CYP3A4 than the wild type allele, which could be caused by a repressor element in the wild type promoter [27, 28]. CYP3A4*1B is associated with poor metabolism of artemether, lumefantrine [29, 30] and quinine [31].

The expression of CYP3A5 in the human liver differs vastly between ethnical groups and within the groups. CYP3A5*3 is the most frequently recognized non-functional allele and one of the most frequent polymorphism among cytochrome P450 genes being recognized [32]. In Caucasians, the frequency of CYP3A5*3 has been showed to be ≥90% [33] whilst the occurrence among black Africans differs between 11 and 78% over the African continent [34].

This study aimed at determining presence of CYP 450 variant alleles (CYP2C8*2, CYP2C8*3, CYP2B6*6, CYP3A4*1B and CYP3A5*3) responsible for the metabolism of artemisinin-based combinations employed in the treatment of uncomplicated malaria and complicated malaria (injectable artesunate) in Tanzania where malaria is endemic.

## Methods

### Study area and subjects

This was a cross-sectional study conducted from April 2013 to April 2014 at Karume Health Centre in Igombe, a semi-urban and malaria meso-endermic area in Ilemela district, Mwanza region, Tanzania, with a population of about 40,000.

### Sampling procedure

Serial sampling method was employed where every third and sixth individual was selected until the sample size was reached.

### Blood sample collection and DNA extraction

Venous blood (1-2 ml) was collected into sterile EDTA-containing tubes from 256 healthy volunteers after obtaining informed consent from each of the participants. The collected blood was stored at -40°C until genomic DNA extraction was done. Genomic DNA extraction was performed with Gene JET Genomic DNA Purification Kit (Thermo Scientific) according to the manufacturer’s instructions.

### Genotyping

#### PCR conditions

The PCR master mix contained 2 μl 1X Dream Taq Green Buffer (Fermentas), 2 μl 1 mM dNTP Mix (Thermo Scientific), 10 μl 0.5 μM reverse primer (Eurofins), 10 μl 0.5 μM forward primer (Eurofins), 0.25 μl 1.25 U Dream Taq (Thermo Scientific).

PCR amplification was done using primers shown in Table 1 and conditions indicated in Table 2: 2.5% agarose gel electrophoresis was performed to confirm successful PCR amplification for all samples.

**Table 1:** **Primers used**

SNP	Primers		Reference
	Forward	Reverse	
CYP3A4*1B	5′-ATGGCCAAGTCTGGGATGAG-3′	5′-CTCACCTCTGTTCAGGGAAAC-3′	[29, 35]
CYP2B6*6	5-′TGAGTGATGGCAGACAATCACA-3′	5-′CAAGTTGAGCATCTTCAGGAACT-3′	[29, 35]
CYP3A5*3	5′-TGGAGAGTGGCATAGGAGATAC-3′	5′CCATACCCCTAGTTGTACGACACA-3′	[29, 35]
CYP2C8*2	5′-GAACACCAAGCATCACTGGA-3′	GAAATCAAAATACTGATCTGTTGC-3′	[36]
CYP2C8*3	5′-CTAAAGGACTTGGTAGGTGCA-3′	5′-CAGGATGCGCAATGAAGACC-3′	[29, 35]

**Table 2:** **PCR conditions employed for various SNPs**

SNP	PCR conditions					
	Initial denaturation	Denaturation	Annealing	Extension	Final extension	Hold temp
CYP3A4*1B	94°C for 2 min	94°C for 30 s (30 cycles)	60°C for 30 s (30 cycles)	72°C for 90 s (30 cycles)	72°C for 5 min	4°C
CYP2B6*6	96°C for 3 min	96°C for 30 s (40 cycles)	64°C for 1 min 30 s (40 cycles)	72°C for 1 min 30 s (40 cycles)	72°C for 10 min	4°C
CYP3A5*3	96°C for 3 min	96°C for 30 s (40 cycles)	59.3°C for 30 s (40 cycles)	72°C for 30 s (40 cycles)	72°C for 10 min	4°C
CYP2C8*2	96°C for 3 min	96°C for 30 s (40 cycles)	56°C for 1 min (40 cycles)	72°C for 1 min (40 cycles)	72°C for 10 min	4°C
CYP2C8*3	96°C for 3 min	96°C for 30 s (30 cycles)	64°C 1 min 30 s (30 cycles)	72°C 1 min 30 s (30 cycles)	72°C for 10 min	4°C

### Restriction fragment length polymorphism (PCR-RFLP) for CYP2C8*2 and CYP2C8*3

The PCR product (10 μL) was digested with ten units of **Bcll** (for CYP2C8*2) in Buffer G (Thermo Scientific) at 55°C for three hours or five units of **BseRI** (for CYP2C8*3) in CutSmart Buffer (New England Biolabs) at 37°C for 15 min. Digestion was followed by thermal inactivation of BcII at 80°C for 20 min. Five μL of inactivated digested product mixed with a drop of loading dye was analysed by electrophoresis on a 2.5% agarose gel containing ethidium bromide and visualized under UV light illuminator. The mutant variant of CYP2C8*2 was undigested and observed as a band of 107 bp, while wild type DNA was observed as bands of 57 and 50 bp after Bcll digestion. Results with bands at 107, 57 and 50 bp were recorded as heterozygotes. For CYP2C8*3, wild type DNA was observed as bands of 187 and 282 bp after BseRI digestion. Mutants were undigested with one band at 439 bp and heterozygotes yielded bands of 157,282 and 439 bp.

### CYP3A4*1B, CYP3A5*3 and CYP2B6*6 sequencing

The PCR products were purified from excess dNTP and primers. To every PCR sample of 15 μl, 7.5 μl of ExoSAP Mix was added consisting of 0.019 μl Exonuclease I (20 U/μl), 0.190 μl SAP (Shrimp Alkaline Phosphate 1 U/μl) and 7.290 μl of sterile water. The samples were incubated at 37°C for 30 min and then at 90°C in a thermocycler for 5 min. In order to prepare for sequencing 2 μl of the PCR-product was mixed with 0.4 pmol of the primer and 15.6 μl of sterile water. The samples were then sent for sequencing at Uppsala Genome Centre after DNA quantification.

### Ethical clearance

Ethical and study approval was granted by the joint Catholic University of Health and Allied Sciences (CUHAS)/Bugando Medical Centre (BMC) Institutional Review Board. All participants signed a written informed consent.

### Statistical analysis

Allelic frequencies were analysed using SPSS 17, SNPs analysed were evaluated using the online tool Hardy-Weinberg equilibrium calculator including analysis for ascertainment bias [37].

## Results

A total of 256 subjects were studied for the frequency of SNPs in this study. The median age of the subjects was 28 (IQR 22-35) years and 80.5% (206/256) were females. All study subjects were natives. Successfully amplified samples were 239 for CYP2C8*2 out of 256, 92 for CYP3A4*1B out of 100, 97 for CYP3A5*3 out 100, 100 for CYP2C8*3 out of 100 and 95 for CYP2B6*6 out of 100. For CYP3A4, the A392G mutation known as CYP3A4*1B was investigated. The allele prevalence was found to be 78% among the 92 subjects included in the study, where 57 (62%) of them were homozygote for the mutant variant. The allele prevalence of the A6986G mutation in CYP3A5, named CYP3A5*3 was 16% among 97 subjects. Out of the 97 subjects, only two (2%) were homozygous for the mutation.

For CYP2C8 the A805T mutation known as CYP2C8*2 was investigated. The allele prevalence was found to be 19% among the 239 subjects included in the study, where 11 (4.6%) of them were homozygote for the mutant variant. The G416A mutation (CYP2C8*3) was also analysed, the allele prevalence was identified to be 10% among the 100 subjects included in the study, where 0 (0%) were homozygote for the mutant variant. For CYP2B6, the G15631T mutation (CYP2B6*6) was investigated. The allele prevalence was found to be 36% among the 95 subjects included in the study, where 11 (11.6%) were identified as homozygote for the mutant variant. The allele frequencies were all at Hardy Weinberg equilibrium. These results are in agreement with several earlier studies from other African countries (Tables 3 and 4).

**Table 3:** Allele frequency of CYP3A4*1B and CYP3A5*3 reported from previous studies in African populations

*Country*	*Number of subjects*	CYP3A4*1B (%)	CYP3A5*3 (%)
Senegal	178	79 [38]	-
Cameroon	72	78.0 [39]	-
East Africa		-	36 [40]
South Africa	980,987	73.0-76.0 [41, 42]	15.0 [41]
Ghana	129-204	72.0-81 [38, 43]	15.0 [43]
Mozambique	86	73.8 [44]	-
Sao Tomé and	78	67.7 [44]	-
Tanzania (Zanzibar)	99, 92	69.2 [45]	15.8 [45]
Tanzania (Mainland)	165	74 [29]	18 [29]
Guinea Bissau		74 [46]	-
Zimbabwe	100	-	77.6 [47]

**Table 4:** Allele frequencies of CYP2C8*2, CYP2C8*3 and CYP2B6*6 reported from previous studies in African populations

*Country number*	CYP2C8*2 (%)	CYP2C8*3 (%)	CYP2B6*6 (%)
Burundi 60	-	-	50.0 [48]
Burkina Faso 275	15.5 [49]	0.3 [49]	-
Ghana 200	17-17.9 [50–52]	0 [50]	46.9 [22]
Zimbabwe 74	-	-	49 [53]
Mozambique 115	16.0 [54]	-	-
Senegal 88	22.0 [36]	-	-
Tanzania 165 (Zanzibar)	13.9 [55]	2.1 [55]	-
Tanzania 165 (Mainland)	-	-	34-41.8 [29, 56]
South Africa 182 (Xhosa)	-	-	32.14 [57]
Madagascar 153	15.0 [36]	-	-

## Discussion

The present study was designed to establish the frequencies of common CYP polymorphisms in Mwanza, Tanzania, as a background for future studies on drug metabolism in clinical trials with anti-malarial drugs. Since artemisinin-based combination therapy (ACT) is the recommended treatment, the study focused on the markers that have been shown to be mostly related with metabolism of anti-malarial drugs, taking into account that some of these mutations have been shown to affect therapeutic response and safety during treatment.

In general, there were no big differences in allele frequencies in comparison with other studies done on African populations (Tables 3 and 4). Despite there being limited information in Tanzania, a few studies on CYP markers done have been performed in Zanzibar, where the population history is different from mainland Tanzania, and Mwanza is geographically distant from Zanzibar. It cannot thus be taken for granted that genetic polymorphisms should be the same in these locations.

This study identified similar findings to Zanzibar in the CYP3A5*3 variant allele: 16 vs 15.8% [45], and for CYP2C8*3 where none of the patients was homozygous mutant [55]. However the study recorded 10% heterozygous CYP2C8*3 which differs from the 0% recorded from earlier studies in Zanzibar [55] and a slightly higher CYP2C8*2 (19%) compared to 13.9% recorded in Zanzibar [55].

This study identified that 4.6% of participants were homozygotes for the slow metabolizer allele (CYP2C8*2) representing a significant population at high risk to amodiaquine toxicity due to amodiaquine accumulation, taking into account artesunate-amodiaquine is an alternative to artemether-lumefantrine in the treatment of uncomplicated malaria in Tanzania and other sub-Saharan African countries [1, 58].

The mutation A6986G leads to splicing defects in intron 3, introducing a stop codon causing severely decreased enzyme activity [25]. The homozygous mutants of CYP3A5*3 was detected in 2% of the study subjects suggesting poor metabolism to anti-malarial drugs such as quinine, quinidine, artemether, chloroquine, halofantrine, lumefantrine, mefloquine, primaquine, artemisinin, arteether, arterlinic acid and proguanil [1], which suggests that a significant proportion of the population is exposed to toxicity of these anti-malarial drugs, including ACT, such as artemether-lumefantrine and artesunate-mefloquine during treatment taking into account that there is a high diversity in CYP3A5 in Africa.

CYP3A5 diversity is higher in East Africa [6, 40], which has higher frequencies than other parts of sub-Saharan Africa [6]. The diversity explains a difference between CYP3A5*3 data from this study and that observed from other areas of East Africa [40], but is similar to data from other parts of Tanzania mainland [29] and South Africa [41]. A high frequency of CYP2B6*6 homozygous mutant allele from this study indicates that a significant proportion of the population are poor metabolizers of artemisinin and arteether [1] resulting in an increased risk to toxicity of the above anti-malarial drugs.

For CYP3A4*1B, a very high proportion of the poor metabolizer allele (PM) in the population indicates high risk to artemisinin, artemether and lumefantrine toxicity due to a less efficient metabolism as discussed with CYP3A5*3 and CYP2C8*2 above. The high frequency recorded in this study demonstrates the previous documentations on a higher occurrence of this variant allele in Africans than Caucasians [59]. The high allele frequency observed from the study population is similar to other African populations, Ghana being one example [43]. Population variability for CYP3A4 is also exceptionally high, more than 100-fold [60]. The frequency of the CYP3A4*1B mutation differs between populations. Among Caucasians and Hispanics the frequency is 3-5%, among Asians it seems to be absent [60] while the variant is more common among Africans as shown by findings from this study and previous studies (Tables 3, 4 and 5).

**Table 5:** Allele and genotype frequencies of CYP3A4*1B and CYP3A5*3 in Igombe, Tanzania, and expected frequencies according to Hardy Weinberg equilibrium

Gene	SNP	Allele frequency	Genotype frequency			
			wt/wt	wt/mut	mut/mut	Gene allele
CYP3A4	A392G	G, 78%	5.4% (5/92)	32.6% (30/92)	62% (57/92)	CYP3A4*1B wild type
		A, 22%				
Expected			4	31	56	
CYP3A5	A6986G	G, 16%	70.1 (68/97)	27.9% (27/97)	2% (2/97)	CYP3A5*3 wild type
		A, 84%				
Expected			68	26	2	
CYP2C8	G416A	A, 10%	80% (80/100)	20% (20/100)	0% (0/100)	CYP2C8*3 wild type
		G, 90%				
Expected			81	18	1	
CYP2C8	A805T	T, 19%	66.1 (158/239)	29.3% (70/239)	4.6% (11/239)	CYP2C8*2 wild type
		A, 81%				
Expected			159	74	9	
CYP2B6	G15631T	T, 36%	39% (37/95)	49.4% (47/95)	11.6% (11/95)	CYP2B6*6 wild type
		G, 64%				
Expected			39	44	12	

Note: wt/wt means homozygous wild type, wt/mut means heterozygous mutant and mut/mut means homozygous mutant.

CYP2C8*3 has been shown to affect metabolism to a higher extent than CYP2C8*2. This study did not identify any homozygous patients who are PM of amodiaquine. This finding is not surprising since this variant allele has been shown to be rare/uncommon among indigenous Africans and predominantly concentrated in Caucasians [14]. CYP2C8*3 frequencies recorded in this study are similar to Uganda [61] and other African countries [50].

Findings from this study demonstrate the existence of diverse genetics of the above CYP 450 s in Tanzania, thus calling for an evaluation of the impact of CYP 450 polymorphisms on metabolism of artemisinin-based combinations and its impact on clinical outcome and toxicity among *P. falciparum* malaria-infected patients in African populations.

## Conclusion

Prevalence of CYP2C8*2, CYP3A5*3, CYP3A4*1B and CYP2B6*6 mutations are common while CYP2C8*3 mutations are rare in a Tanzanian population. The frequencies recorded in this study are comparable to data obtained from previous studies on African populations. Pharmacogenomics data, such as that presented in this paper, provides a basis for further studies on impact of polymorphism in ACT safety and efficacy.
